# 

**Published:** 1868-04

**Authors:** 


We recently promised to present an article through the pages of the Register, on the subject of taking impressions; and now we hope some body will assist us, by sending us the article and we will print it; a proper division of labor is all we ask.

---

The second edition of Taft's Operative Dentistry is out, and came to hand a few days ago. T.

The next No. of the Register will contain the proceedings of the Ohio Dental College Association. Also the minutes of the Association of the Colleges of Dentistry recently held in New York.

OF THE WEST.

*Issued Monthly, at $3 a Year in Advance.*

DEVOTED TO THE INTERESTS OF THE DENTAL PROFESSION.

EDITED BY J. TAFT AND G. WATT.

The volume commences in January and closes in December.

The Register being issued monthly is always fresh, therefore indispensable to the practitioner who desires to keep posted up with the new inventions and improvements which are daily developed. Neither expense nor effort will be spared to give value and variety to its contents. Articles will be illustrated with well executed engravings whenever they will add to the interest or assist in explanation.

Its extensive circulation and frequency of issue makes it one of the BEST DENTAL ADVERTISING MEDIUMS in the United States.

RATES OF ADVERTISING.

One page, 1 year, $50. Half page, 1 year, $30. Quarter page, or card, 1 year $20.

All advertising bills payable in advance, or at furthest after the issue of the first number containing the advertisement. All transient advertisements to be paid for in advance.

☞ CONTRIBUTIONS SOLICITED.

Address, J. TAFT, or "DENTAL REGISTER," Cincinnati Ohio.

Business Communications to

J. TAFT, Publisher,

No. 238 Fourth St., between Plum and Central Avenue

